# Transcatheter edge-to-edge repair and left ventricular assist devices for secondary mitral regurgitation in advanced heart failure: a scoping review

**DOI:** 10.1186/s13019-026-04245-z

**Published:** 2026-05-07

**Authors:** Rida Shakeel, Tuba Basit, Sohaib Aftab Ahmad Chaudhry, Huzaifa Sabir Nawaz, Amal Tahir, Syeda Masooma Jafri, Diksha Ladhani, Rachna Katyara, Muhammad Hussain, Muhammad Khalid Afridi, Raghabendra Kumar Mahato, Muhammad Aqib Faizan

**Affiliations:** 1 Department of Surgery, Dow Medical College, Karachi, Pakistan; 2 Department of Medicine, ABWA Medical College, Faisalabad, Pakistan; 3 Department of Neurosurgery, Services Hospital, Lahore, Pakistan; 4 Fellowship Interventional Cardiology, Head of Cardiology Department, Abwa Hospital & Research Center, Faisalabad, Pakistan; 5 Department of Medicine, Dow Medical College, Karachi, Pakistan; 6 Department of Medicine, Gandaki Medical College Teaching Hospital, Pokhara, Nepal; 7 Department of Medicine, Gomal Medical College, Dera Ismail Khan,, Pakistan

**Keywords:** Transcatheter edge-to-edge repair, Left ventricular assist device, Secondary mitral regurgitation, Advanced heart failure, Guideline-directed medical therapy

## Abstract

**Background:**

Secondary mitral regurgitation (SMR) worsens outcomes in advanced heart failure. Transcatheter edge-to-edge repair (TEER) and left ventricular assist device (LVAD) implantation represent advanced treatment options for patients with persistent symptoms despite guideline-directed medical therapy (GDMT).

**Methods:**

A scoping review using a PICO framework identified contemporary studies published up to 2025 involving adults with advanced heart failure (NYHA III–IV, INTERMACS 1–7) and ≥moderate SMR. Outcomes included mortality, heart failure hospitalizations, functional status, mitral regurgitation reduction, ventricular remodeling, complications, and quality of life.

**Results:**

Nine studies including over 2,000 patients were analyzed (mean age 60–72 years; LVEF 15–33%). TEER was associated with lower mortality and fewer heart failure hospitalizations compared with GDMT in selected patients. LVAD recipients generally had more advanced disease (predominantly INTERMACS 1–3) with higher early mortality but substantial functional improvement. TEER achieved MR ≤ 2 + in 84–99% of patients with low procedural complication rates (1–9%), whereas LVAD therapy demonstrated greater functional improvement but higher device-related complications.

**Conclusion:**

TEER is associated with improved outcomes in selected patients with advanced heart failure and SMR, while LVAD therapy remains essential for patients with end-stage disease requiring durable circulatory support.

**Supplementary Information:**

The online version contains supplementary material available at 10.1186/s13019-026-04245-z.

## Highlights

TEER reduces mitral regurgitation and heart failure hospitalizations in selected patients.

LVAD provides durable circulatory support in end-stage heart failure.

TEER is associated with lower procedural complication rates.

LVAD recipients demonstrate greater functional improvement but higher device complications.

Direct comparative evidence between TEER and LVAD remains limited.

**Supplementary Information:**

The online version contains supplementary material available at 10.1186/s13019-026-04245-z.

## Introduction

Heart failure with reduced ejection fraction (HFrEF) remains a prevalent and high-mortality condition despite major therapeutic advances. There are over 64 million people living with heart failure around the world, and approximately 32 million have HFrEF [1]. Despite advances in pharmacologic and device-based therapies, nearly half of patients die within five years of diagnosis, highlighting the continued need for improved management strategies beyond conventional medical and device therapy [2].

Current treatments for HFrEF include guideline-directed medical therapy (GDMT), including angiotensin receptor-neprilysin inhibitors and sodium-glucose cotransporter-2 inhibitors as well as implantable cardioverter-defibrillators (ICDs). However, many patients progress to advanced stages of the disease and require more invasive treatment options [3].

A common consequence of HFrEF is secondary mitral regurgitation (SMR), also referred to as functional mitral regurgitation. SMR frequently develops in advanced HFrEF due to LV remodeling and is strongly associated with worse symptoms and prognosis. SMR can also be identified and quantified by echocardiography with the assessment of regurgitant volume, the effective regurgitant orifice area, and the degree of left-ventricular remodeling [4].

Recommendations for first-line therapies for SMR in patients with HFrEF include guideline-directed medical therapy (GDMT) for the optimization of heart function and cardiac resynchronization therapy (CRT), a device-based therapy designed to restore synchronized ventricular contraction. Optimization of GDMT and CRT is recommended, but many patients with stage D disease remain symptomatic and require advanced therapies.

Left ventricular assist devices (LVADs) and transcatheter edge-to-edge repair (TEER) represent two advanced therapeutic strategies used in patients with advanced heart failure. LVADs are mechanical pumps implanted to help the heart circulate blood and are used either as a bridge to transplant or for long-term therapy in patients not eligible for transplant. Contemporary LVAD systems utilizing fully magnetically levitated centrifugal-flow pumps have demonstrated improved survival and reduced device-related complications compared with earlier axial-flow devices, as shown in the MOMENTUM-3 trial [5]. TEER is a type of minimally invasive surgical procedure that involves the use of a device, such as MitraClip, to clip the leaflets of the mitral valve together and thus reduce regurgitation. TEER has emerged as an important option for patients who are not suitable candidates for surgical mitral valve repair due to advanced disease or high surgical risk. The COAPT trial demonstrated that TEER reduced heart failure hospitalizations and improved survival compared with medical therapy in carefully selected patients meeting strict inclusion criteria [6].

These findings have transformed the treatment approach in heart failure patients with significant mitral valve regurgitation. However, the MITRA-FR study has shown that precise and proper patient selection is critical for optimal patient outcomes, specifically by considering the severity of mitral valve regurgitation and the left ventricular dimensions [7]. Most recently, further research including the RESHAPE-HF2 study indicated that TEER is superior to medical therapy for the treatment of patients with heart failure and moderate mitral valve regurgitation and will decrease hospital readmission [8].

Importantly, these therapies are typically applied in different clinical contexts and stages of disease, and therefore should not be interpreted as directly comparable interventions. However, there is currently limited evidence directly comparing these two modalities, and most of the data comes from small, non-randomized studies with inconsistent designs, making it difficult to identify the best treatment strategy for specific patients. Additionally, there are no standardized outcome measures that will allow us to synthesize across studies.

Given these limitations, there is a need for a scoping review. Our scoping review would provide an overview of the current research in this area, summarizing findings related to key patient-centered outcomes such as survival, symptom burden, and functional status, identify knowledge gaps in the evidence base as well as next steps for future research. Additionally, a scoping review could help analyze the existing evidence base to inform the development of better individualized treatments, which may increase patient outcomes and ultimately improve the care of HFrEF in clinical practice.

## Methods

### Objective and review question

This scoping review aims to analyze and systematically map the observational and randomized evidence reported in contemporary studies comparing LVAD implantation and TEER in patients with advanced HFrEF and secondary mitral regurgitation (SMR). The objective of this review is to explore reported clinical, echocardiographic and patient-centered outcomes associated with these interventions, including mortality, heart failure hospitalization, functional capacity, ventricular remodeling, procedural safety, and quality of life. A scoping review methodology was selected due to the limited availability and heterogeneity of the evidence, allowing broad mapping of existing research and identification of gaps.

### Study design

A scoping review framework was chosen due to its flexibility in mapping heterogeneous evidence. Our review was guided by the Arksey and O’Malley (2005) framework, refined by Levac et al. (2010), encompassing the following stages: identifying relevant studies, selecting eligible studies, charting data, and collating and reporting results. The review process and reporting adhered to the 2018 Preferred Reporting Items for Systematic Reviews and Meta-Analysis extension for Scoping Reviews (PRISMA-ScR) guidelines to ensure methodological transparency and reproducibility.

### Inclusion and exclusion criteria

Eligible studies included observational designs (prospective or retrospective cohort studies, registry-based analyses or case-control studies) including adults (≥ 18 years) with advanced HFrEF and secondary mitral regurgitation. Studies were included if they directly compared LVAD implantation versus TEER or reported outcomes of either intervention in this patient population. Outcomes of interest included clinical endpoints (mortality, heart failure rehospitalization), echocardiographic measures (MR reduction, left ventricular remodeling), functional outcomes (NYHA class, 6MWT), procedural and device-related complications, and quality-of-life metrics. Studies were excluded if they were case reports or case series with fewer than 10 patients, review articles, conference abstracts without primary data, or non-human or pediatric studies. Studies focusing on primary/degenerative MR, non-HFrEF populations, or those lacking outcomes relevant to LVAD or TEER were also excluded. Only full-text articles published in English were included. Full inclusion and exclusion criteria are provided in *Supplementary Table 2.*

### Search strategy

A comprehensive literature search of relevant studies from PubMed, Cochrane, Embase, Scopus, and ClinicalTrial.gov databases was conducted from inception to September 2025. The search combined Medical Subject Headings (MeSH) and Emtree terms with relevant free-text keywords related to heart failure, secondary mitral regurgitation (SMR), LVAD and TEER (mitraclip) using Boolean operators. The search strategy was developed in consultation with the study team to ensure comprehensive identification of relevant literature. Reference lists of included articles and previous relevant reviews were screened manually to identify additional eligible studies. The full search strategy for each database is provided in *Supplementary Table 1.*

### Study selection

**Fig. 1:**
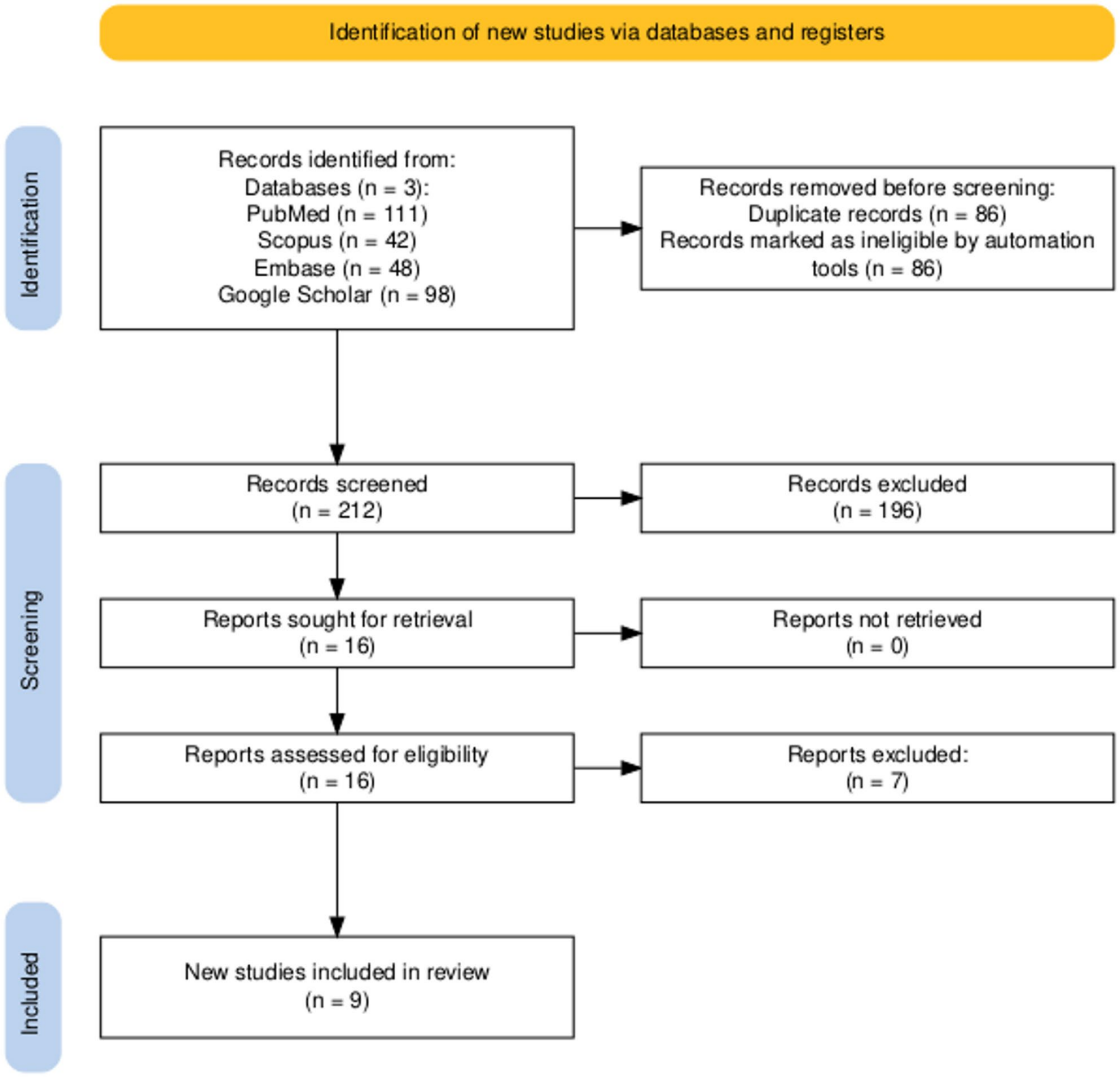
PRISMA Flowchart

All identified citations were imported into reference management software, and duplicates were removed systematically. Two independent reviewers (D.L and T.B) screened titles and abstracts using Rayyan systematic review software to identify studies meeting the eligibility criteria. Full text articles of potentially eligible studies were retrieved and assessed against the inclusion criteria. Discrepancies were resolved through discussion or consultation with a third reviewer. A PRISMA flow diagram was generated to document the selection process, illustrating the number of records identified, screened, included, and excluded (Fig. 1). As recommended for scoping reviews, formal risk-of-bias assessment was not performed because the aim of this review was to map the available evidence rather than estimate pooled treatment effects.

### Data extraction

Data were extracted independently by two reviewers (A.T. and R.K.) using a standardized Microsoft Excel spreadsheet. Extracted information included study characteristics (first author, publication year, country, study design, and sample size), patient demographics (age, sex, baseline left ventricular ejection fraction, mitral regurgitation severity, and NYHA class), and details of interventions and comparators (type of LVAD device, TEER system used, and procedural characteristics). Outcomes collected included mortality, heart failure hospitalization, MR reduction, left ventricular remodeling, functional improvement, procedural complications, and quality-of-life measures, when reported. Discrepancies in data extraction were resolved through discussion and consensus with a third reviewer (R.S) to ensure accuracy and consistency.

### Data analysis

Due to heterogeneity in study design, patient populations, and outcome reporting, a narrative synthesis approach was employed rather than quantitative meta-analysis. Data were systematically summarized and organized into clinical, echocardiographic and patient-centered outcomes, including mortality, heart failure hospitalization, left ventricular remodeling, and quality of life. The synthesis focused on identifying consistent trends, gaps in evidence, and variations in reported outcomes between LVAD implantation and TEER in patients with advanced heart failure and secondary mitral regurgitation (SMR).

### Ethical considerations

As this study analyzed previously published data, no ethical approval or patient consent was required. All procedures adhered to the ethical principles of systematic review conduct and reporting, ensuring transparency and data integrity.

### Results

### Baseline characteristics

Patients enrolled in the included studies had advanced heart failure with predominantly NYHA class III–IV symptoms. Those undergoing transcatheter edge-to-edge repair (TEER) typically had less severe hemodynamic compromise, with most having INTERMACS profiles 4–7 and lower dependence on inotropes, whereas patients selected for left ventricular assist device (LVAD) implantation were more critically ill, with INTERMACS profiles 1–3 and frequent inotrope requirement (Tables 1 and 2). Baseline secondary mitral regurgitation was moderate-to-severe to severe in most TEER cohorts, whereas LVAD cohorts demonstrated more variable degrees of mitral regurgitation ranging from mild to severe (Table 2). Mean age ranged from 60 to 72 years, and the study populations were predominantly male. Detailed comorbidity prevalence and background medical therapy are provided in Table 2.

**Table 1:** Characteristics of studies included in the scoping review

Author	Year	Country / Centers	Study Design	Sample Size (*N*)	Groups (Intervention vs. Control)	Age (mean/median ± SD)	Male *n* (%)	Female *n* (%)
Kreusser M.M.	2020	Germany	Retrospective	20	LVAD	NA	NA	NA
				17	LVAD + MC	NA	NA	NA
Mehra M. R.	2022	69 US centers	Extended-phase study protocol	515	Magnetically levitated centrifugal-flow pump	60.67 ± 11.89	410 (79.6)	105 (20.4)
				505	Axial-flow pump	62.0 ± 11.15	413 (81.8)	92 (18.2)
Frea S.	2024	Turin, Italy	Prospective single-center study	27	Medical therapy	60 ± 12	21 (78)	6 (22)
				36	TEER	65 ± 10	31 (86)	5 (14)
Kumbhani D. J.	2024	NA	Randomized Controlled Trial	312	GDMT	72 (mean)	63%	37%
				302	Mitraclip + GDMT	NA	NA	NA
Hahn R. T.	2020	NA	Randomized Controlled Trial	> Mod TR: 54 < Mild TR: 246	GDMT	NA	NA	NA
				> Mod TR: 44 < Mild TR: 255	Mitraclip + GDMT	NA	NA	NA
Stone G. W.	2023	New York, USA	Randomized Controlled Trial	312	GDMT	72.8 ± 10.5	192 (61.5)	120 (38.5)
				302	TEER	71.7 ± 11.8	201 (66.6)	101 (33.4)
Fox H.	2021	Germany	Observational prospective	27	HM3	64 ± 5.5	25 (93)	2 (7)
				27	TMR + HM3	63 ± 9.1	23 (85)	4 (15)
Valente S	2024	Italy	Review article	NA	Momentum 3 trial	60.3 ± 12.3	79.6%	20.4%
				NA	COAPT trial	71.7 ± 11.8	66.6%	33.4%
				307	MITRA-FR trial	71.7 ± 11.8	78.9%	21.1%
Pausch J.	2022	Germany	Retrospective	46	LVAD	64	81.6%	18.4%
				21	TEER + LVAD	56	NA	NA

AF = atrial fibrillation; GDMT = guideline-directed medical therapy; HM3 = HeartMate 3; LVAD = left ventricular assist device; MC = mitral clip; NA = not available; TEER = transcatheter edge-to-edge repair; TMR = transcatheter mitral repair; TR = tricuspid regurgitation

**Table 2:** Baseline patient characteristics, comorbidities, functional status, laboratory values, and echocardiographic parameters in the included studies

Study & Group	INTERMACS Profile (mean or distribution)	NT-proBNP (pg/mL)	Creatinine (mg/dL)	Hemoglobin (g/dL)	Key Comorbidities (%)	NYHA Class Distribution	6MWT (m)	LVEF (%)	MR Severity	LV Volumes (mL)	RVSP (mmHg)	Selected Medications (%)
Kreusser M.M. 2020												
LVAD	5: 40%; 6: 20% (full distribution in footnote)	13,926 ± 16,255	1.21 ± 0.44	NA	NA	III: 45%; IV: 55%	276 ± 113	15.3 ± 7.2	2+: 65%; 3+: 35%	NA	NA	NA
LVAD + MC	6: 35%; 7: 29% (full in footnote)	8,112 ± 6,164	1.21 ± 0.44	NA	NA	III: 53%; IV: 29%	378 ± 114	16.2 ± 9.3	3+: 82%	NA	NA	NA
Mehra M. R. 2022												
Centrifugal-flow pump	1–2: 33%; 3: 53%	NA	1.3 ± 0.45	NA	DM: 45; Prior stroke: 10; CKD/AF: 42	NA	NA	NA	Mod/severe: 42	NA	NA	Inotrope: 86; Diuretic: 85; BB: 55; ACEi/ARB: 31
Axial-flow pump	1–2: 32%; 3: 50%	NA	1.32 ± 0.40	NA	DM: 44; Prior stroke: 11; CKD/AF: 47	NA	NA	NA	Mod/severe: 45	NA	NA	Inotrope: 83; Diuretic: 91; BB: 53; ACEi/ARB: 34
Frea S. 2024												
Medical therapy	4: 74%	10,023 ± 8,169	1.7 ± 0.8	12.3 ± 2.1	NA	NA	NA	21 ± 4	NA	LVEDV: 258 ± 93	NA	BB: 85; ACEi/ARB/ARNI: 44
TEER	4: 64%	10,321 ± 5,467	1.5 ± 0.5	11.8 ± 2.1	NA	NA	NA	21 ± 3	NA	LVEDV: 241 ± 98	NA	BB: 90; ACEi/ARB/ARNI: 25
Kumbhani D. J. 2024												
GDMT	NA	NA	NA	NA	NA	II/III/IV	NA	31.3	NA	NA	NA	BB: 91; ACEi/ARB/ARNI: 66; MRA: 50
Stone G. W. 2023												
GDMT	NA	5,944 ± 8,438	NA	NA	HTN: 80; DM: 39; AF: 53; Anemia: 63	II: 35%; III: 54%; IV: 11	NA	31.3 ± 9.6	3+: 55%; 4+: 45	LVEDV: 191 ± 73; LVESV: 134 ± 60	44.6 ± 14.0	NA
TEER	NA	5,174 ± 6,567	NA	NA	HTN: 81; DM: 35; AF: 57; Anemia: 60	II: 43%; III: 51%; IV: 6	NA	31.3 ± 9.1	3+: 49%; 4+: 51	LVEDV: 194 ± 69; LVESV: 136 ± 56	44.0 ± 13.4	NA
Fox H. 2021												
HM3	Mean 2.7 ± 0.83	NA	1.5 ± 0.49	NA	HTN: 74; DM: 37; Stroke: 4	NA	NA	22 ± 6	2.3 ± 0.5	LVEDV: 267 ± 141; LVESV: 198 ± 107	NA	NA
TMR + HM3	Mean 2.5 ± 0.94	NA	1.5 ± 0.55	NA	HTN: 67; DM: 30; Stroke: 33	NA	NA	19 ± 5	2.3 ± 0.7	LVEDV: 317 ± 100; LVESV: 246 ± 86	NA	NA
Valente S. 2024												
Momentum 3 trial	INTERMACS 1–4: 99%	NA	NA	NA	NA	NA	NA	17.1 ± 5.0	42%	NA	NA	Inotrope: 87; ACEi/ARNI: 31; BB: 60; Diuretic: 88
COAPT trial	INTERMACS 1–4: 6%	5,174 ± 6,567	NA	NA	NA	III: 54%	NA	31.3 ± 9.1	100%	NA	NA	ACEi/ARNI: 72; BB: 91; Diuretic: 89; MRA: 51
MITRA-FR trial	INTERMACS 1–4: 9%	4,048 ± 3,606	NA	NA	NA	III: 54%; IV: 9%	NA	33.3 ± 6.5	100%	NA	NA	ACEi/ARNI: 82; BB: 88; Diuretic: 99; MRA: 57
Pausch J. 2022												
LVAD	NA	NA	NA	NA	NA	NA	NA	18	NA	NA	NA	NA
TEER + LVAD	NA	NA	NA	NA	NA	NA	NA	20	NA	NA	NA	NA

6MWT = 6-minute walk test; ACEi = angiotensin-converting enzyme inhibitor; AF = atrial fibrillation; ARB = angiotensin receptor blocker; ARNI = angiotensin receptor-neprilysin inhibitor; BB = beta-blocker; DM = diabetes mellitus; GDMT = guideline-directed medical therapy; HM3 = HeartMate 3; HTN = hypertension; INTERMACS = Interagency Registry for Mechanically Assisted Circulatory Support; LVAD = left ventricular assist device; LVEDV = left ventricular end-diastolic volume; LVESV = left ventricular end-systolic volume; MC = mitral clip; MRA = mineralocorticoid receptor antagonist; MR = mitral regurgitation; NA = not available/not reported; NT-proBNP = N-terminal pro-B-type natriuretic peptide; NYHA = New York Heart Association; RVSP = right ventricular systolic pressure; TEER = transcatheter edge-to-edge repair; TMR = transcatheter mitral repair

Kreusser LVAD INTERMACS: full distribution 1:0%, 2:20%, 3:10%, 4:10%, 5:40%, 6:20%, 7:0%

Kreusser LVAD + MC: 1:0%, 2:0%, 3:12%, 4:12%, 5:12%, 6:35%, 7:29%

### Clinical Outcomes

#### Mortality

Mortality outcomes summarized below reflect results reported in the observational studies included in this review and detailed in Table 3. Reported mortality rates represent outcomes at specific study follow-up timepoints rather than cumulative estimates from a single pooled cohort. 1-year all-cause mortality ranged from 17 to 29% in TEER cohorts and 29–54% in LVAD cohorts. 2-year mortality ranged from 27 to 34% after TEER and 17–23% after LVAD in studies reporting this timepoint. 5-year mortality ranged from 57 to 67% in TEER studies comparing TEER with GDMT and from 30 to 51% in LVAD cohorts (Table 3). These differences should be interpreted in the context of substantial baseline differences in disease severity between patient populations.

**Table 3:** Clinical outcomes of the included studies

Author & Year	Intervention / Comparator	30-day Mortality	1-year Mortality	2-year Mortality	Long-term (> 2 years) Mortality	HF Hospitalization / Rehospitalization	NYHA Class Distribution / Improvement	MR Severity Reduction	LV Remodeling / LVEF Change	Procedural / Device-related Complications	KCCQ / PROs / Other Outcomes
Kreusser M.M., 2020	LVAD	NA	29.2%	NA	NA	NA	I:0; II:66.7%; III:25%; IV:8.3%	0:30%; 1:30%; 2:40%; 3:0%	NA	NA	NA
	LVAD + MC	NA	53.7%	NA	NA	NA	I:14.2%; II:42.9%; III:42.9%; IV:0%	0:16.7%; 1:33.3%; 2:50%; 3:0%	NA	NA	NA
Mehra M. R., 2022	Magnetically levitated centrifugal-flow pump	NA	NA	NA	5 years: 30.3%	NA	NYHA I/II at 5 years: 67.9%	NA	NA	Suspected device thrombosis: 2.1%	NA
	Axial-flow pump	NA	NA	NA	5 years: 50.5%	NA	NYHA I/II at 5 years: 69.6%	NA	NA	Suspected device thrombosis: 17.6%	NA
Frea S., 2024	Medical therapy	NA	52%	NA	NA	55%	NA	NA	NA	NA	NA
	TEER	NA	17%	NA	NA	44%	NA	NA	NA	NA	NA
Kumbhani D. J., 2024	GDMT	NA	NA	NA	5 years: 67.2%	67.9% at 24 months	II:63.7%; III:65.5%; IV:85.2%	≤ 2 + at 24 mo: 43.4% (improved ≥ 2+: 15.9%)	LVEF change at 12 mo: -12.8%	NA	KCCQ-OS at 24 mo: 61.2
	Mitraclip + GDMT	NA	NA	NA	5 years: 57.3%	35.8% at 24 months	II:39.7%; III:46.6%; IV:66.7%	≤ 2 + at 24 mo: 99.1% (improved ≥ 2+: 84.1%)	LVEF change at 12 mo: -5.6%	NA	KCCQ-OS at 24 mo: 70.9
Stone G. W., 2023	GDMT	NA	NA	NA	5 years: 67.2%	447 events within 5 years	I:6.5%; II:43.1%; III:67.5%; IV:71.8%	1+:11.1%; 2+:46.8%; 3+:80.8%; 4+:100%	NA	NA	NA
	TEER	NA	NA	NA	5 years: 57.3%	314 events within 5 years	I:14.2%; II:61.2%; III:76.4%; IV:78.7%	1+:69.1%; 2+:94.4%; 3+:98.9%; 4+:100%	NA	Device-specific at 30 days: 1.4%	NA
Fox H., 2021	HM3	1 (3.7%, sepsis)	NA	16.5%	NA	NA	NA	NA	NA	CentriMag use: 8 (30%)	NA
	TMR + HM3	2 (7.4%, MOF)	NA	20%	NA	NA	NA	1 case mild mitral stenosis	NA	CentriMag use: 7 (26%)	NA
Hahn R. T., 2020	GDMT (> Mod TR /	0% / 1.2%	36.6% / 21.0%	63.6% / 39.8%	NA	30d: 16.8%/11.0%; 1y:70.1%/61.1%; 2y:95.4%/78.6%	NA	Persistent moderate-severe TR high	NA	NA	NA
	Mitraclip + GDMT (> Mod TR /	9.1% / 1.2%	23.9% / 18.5%	34.1% / 27.5%	NA	30d:12.6%/12.6%; 1y:59.2%/53.9%; 2y:74.5%/67.2%	NA	Marked reduction in TR severity	NA	NA	NA
Valente S, 2024	Momentum 3 trial	NA	NA	23.1%	NA	NA	NA	NA	NA	NA	NA
	COAPT trial	NA	NA	29.1%	5 years: 57.3%	2 years: 35.8%; 5 years: 33.1%	NA	NA	NA	NA	NA
	MITRA-FR trial	NA	NA	24%	NA	NA	NA	NA	NA	NA	NA
Pausch J., 2022	LVAD	NA	NA	NA	NA	69.6%	NA	NA	LVEF ~ 18%	NA	NA
	TEER + LVAD	NA	NA	NA	NA	100%	NA	NA	LVEF ~ 20%	NA	NA

GDMT = guideline-directed medical therapy; HF = heart failure; HM3 = HeartMate 3; KCCQ = Kansas City Cardiomyopathy Questionnaire; LV = left ventricular; LVEF = left ventricular ejection fraction; MC = mitral clip; MOF = multi-organ failure; MR = mitral regurgitation; NA = not available; NYHA = New York Heart Association; OS = overall summary; PROs = patient-reported outcomes; TEER = transcatheter edge-to-edge repair; TMR = transcatheter mitral repair; TR = tricuspid regurgitation

### Heart failure hospitalization

2- to 5-year cumulative heart failure hospitalization rates ranged from 36 to 45% after TEER versus 55–68% with guideline-directed medical therapy alone **(**Table 3**).** Hospitalization outcomes were less consistently reported in LVAD cohorts; however, unplanned readmissions remain common following LVAD implantation and may occur due to right ventricular failure, recurrent decompensation, arrhythmias, bleeding, infection, or other device-related complications.

### Functional status and quality of life

At 2–5 years, 40–61% of TEER patients and 57–81% of LVAD patients achieved NYHA class I/II. Kansas City Cardiomyopathy Questionnaire scores at two years were higher after TEER than with medical therapy alone (Table 3).

### Mitral regurgitation reduction

TEER achieved MR ≤ 2 + in 84–99% of patients over two years. LVAD implantation was associated with variable degrees of mitral regurgitation reduction depending on ventricular unloading and remodeling (Table 3).

### Left ventricular remodeling

Changes in left ventricular ejection fraction were generally reported at study-specific follow-up intervals ranging from approximately 6 months to 2 years. Across these timepoints, changes in LVEF were minimal after TEER (range − 6 to + 3%) and modest after LVAD (range + 2 to + 10%) (Table 3).

### Complications

Reported complication rates varied across studies and follow-up durations. In TEER cohorts, device- or procedure-related complications were relatively uncommon, ranging from 1 to 9%, and were typically reported during the periprocedural or early follow-up period. In contrast, LVAD cohorts demonstrated higher complication rates (approximately 20–48% at study-specific follow-up), reflecting the invasive nature of device implantation and the severity of underlying disease. Reported LVAD-related complications included pump thrombosis (2–18%) and the need for temporary mechanical circulatory support in approximately 26–30% of selected cohorts, usually referring to short-term perioperative support for hemodynamic instability or right ventricular failure.

### Summary of findings

Across the included studies, TEER was associated with lower short- and mid-term mortality and fewer heart failure hospitalizations compared with guideline-directed medical therapy alone in selected patients with advanced heart failure and secondary mitral regurgitation (Table 3). Both TEER and LVAD were associated with improvements in functional status, with a higher proportion of LVAD recipients achieving NYHA class I/II at longer-term follow-up. TEER consistently achieved reduction of mitral regurgitation to ≤ 2+, whereas mitral regurgitation improvement after LVAD implantation was more variable. Device-related complications were reported more frequently after LVAD implantation than after TEER. Direct head-to-head comparisons between these therapies remain limited.

## Discussion

This scoping review summarizes the available observational evidence regarding TEER and LVAD therapy in patients with advanced heart failure and secondary mitral regurgitation. Although the literature evaluating each intervention individually has expanded, few studies directly evaluate these therapies within comparable patient populations. Importantly, TEER and LVAD are generally applied at different stages of the heart failure disease spectrum and address distinct pathophysiologic mechanisms. Consequently, their clinical use depends on disease severity, hemodynamic status, and therapeutic goals. The purpose of this discussion is therefore to interpret the available evidence, highlight implications for clinical decision-making, and identify remaining gaps rather than to imply direct quantitative comparison between these interventions.

### Mortality and survival outcomes

In selected patients with advanced heart failure and secondary mitral regurgitation, TEER has been associated with improved survival and fewer heart failure hospitalizations compared with guideline-directed medical therapy alone. Evidence from the COAPT trial and subsequent analyses suggests that, in carefully selected patients with less advanced disease, preserved right ventricular function, and lower INTERMACS profiles, correction of secondary mitral regurgitation with TEER may improve clinical outcomes [9–12]. However, these benefits are closely linked to strict patient selection criteria and therefore may not be generalizable to all patients with secondary mitral regurgitation.

In contrast, LVAD therapy is typically reserved for patients with end-stage heart failure (INTERMACS profiles 1–3) who have refractory symptoms despite optimized medical therapy. Contemporary centrifugal-flow devices have demonstrated improved long-term survival and lower rates of device-related complications compared with earlier device generations [13–15]. The higher early mortality reported in LVAD cohorts largely reflects the greater baseline disease severity of these patients rather than a limitation of the therapy itself [13, 16, 17]. Accordingly, outcomes following TEER and LVAD should be interpreted within the context of fundamentally different patient populations and treatment objectives.

### Heart failure hospitalizations and functional status

TEER has been associated with reductions in heart failure hospitalizations by alleviating mitral regurgitation–related volume overload and reducing recurrent decompensation [18]. LVAD therapy may also reduce the burden of hospitalization, particularly in patients receiving bridge-to-transplant or destination therapy; however, comparisons across studies remain challenging due to heterogeneity in patient populations and the frequency of device-related readmissions [13, 19].

These therapies also differ in their impact on functional status. TEER improves symptoms and functional capacity by correcting valvular insufficiency and reducing regurgitant volume, resulting in improvements in NYHA functional class in appropriately selected patients [9, 12]. In contrast, LVAD implantation provides substantial hemodynamic support through mechanical circulatory assistance, which may lead to more pronounced improvements in functional capacity in patients with advanced disease [12, 13]. These differences reflect the distinct mechanisms of the two therapies: TEER primarily reduces mitral regurgitation and associated ventricular volume overload, whereas LVAD therapy provides durable circulatory support for advanced pump failure [16, 18–22].

### Mitral regurgitation reduction and left ventricular remodelling

TEER provides sustained reduction of secondary mitral regurgitation by directly approximating the mitral valve leaflets and reducing regurgitant flow, thereby addressing a key contributor to heart failure progression [9, 23]. In contrast, changes in mitral regurgitation following LVAD implantation occur indirectly and depend on the degree of ventricular unloading and reverse remodeling achieved after device implantation [6]. Consequently, the degree of mitral regurgitation improvement following LVAD therapy is more variable across studies. These differences highlight that TEER and LVAD target distinct components of heart failure pathophysiology and therefore represent complementary rather than interchangeable therapeutic strategies [19, 24].

#### Quality of life and patient-reported outcomes

Both interventions may improve quality of life, although the mechanisms and trade-offs differ. TEER has been associated with clinically meaningful improvements in patient-reported outcomes because of reductions in MR severity, reduced hospitalizations, and enhanced functional status [9]. LVAD therapy is also associated with increased exercise tolerance and improved quality of life which can be overshadowed by device-related issues including infections, bleeding, and ongoing device management, including driveline care, anticoagulation, and monitoring for device-related complications [25, 26]. Therefore, QoL improvements with LVAD therapy are highly dependent on patient expectations and the willingness to accept permanent device dependence.

#### Complications and safety profiles

The minimally invasive strategy of TEER results in an excellent safety profile with a relatively low rate of procedural complications [9, 12]. LVAD implantation is associated with increased perioperative and long-term adverse events such as bleeding, infection and hemocompatibility-related complications. Indeed, devices using centrifugal flow demonstrated a much lower incidence of pump thrombosis and stroke compared with earlier axial-flow systems [13, 16]. These distinct profiles of risk are critical for therapeutic choices in advanced HF.

#### Clinical implications

Clinically, TEER is most appropriate in patients with advanced HF and severe secondary MR who have preserved RV function, manageable pulmonary pressures, and INTERMACS 3–4 profiles. LVAD treatment may be considered in patients with end-stage heart failure, with progressive hemodynamic decline or who have failed less invasive approaches. Candidacy is heavily influenced by RV dysfunction and pulmonary hypertension as significant RV failure or fixed pulmonary vascularity can render either approach ineffective. These data argue in favor of a stepwise treatment strategy, with TEER used to stabilize disease at an earlier stage and LVADs implemented later in the progression to accomplish this.

### Shared decision-making considerations

With the disparate risk–benefit profiles of TEER and LVAD therapy, the importance of shared decision-making cannot be overemphasized. Management discussions should be guided by frailty, burden of comorbidity, expected life span and tolerance for device-related disruption of lifestyle. Whereas TEER may better serve symptomatic relief and reduction of procedural risk, LVAD therapy may be considered for patients who are more concerned with survival and improvement despite a higher long-term burden. Multidisciplinary HF team engagement and systematic goals-of-care conversations are crucial to ensure care delivery is consistent with the values of patients.

### Limitations, gaps, and future directions

**Fig. 2:**
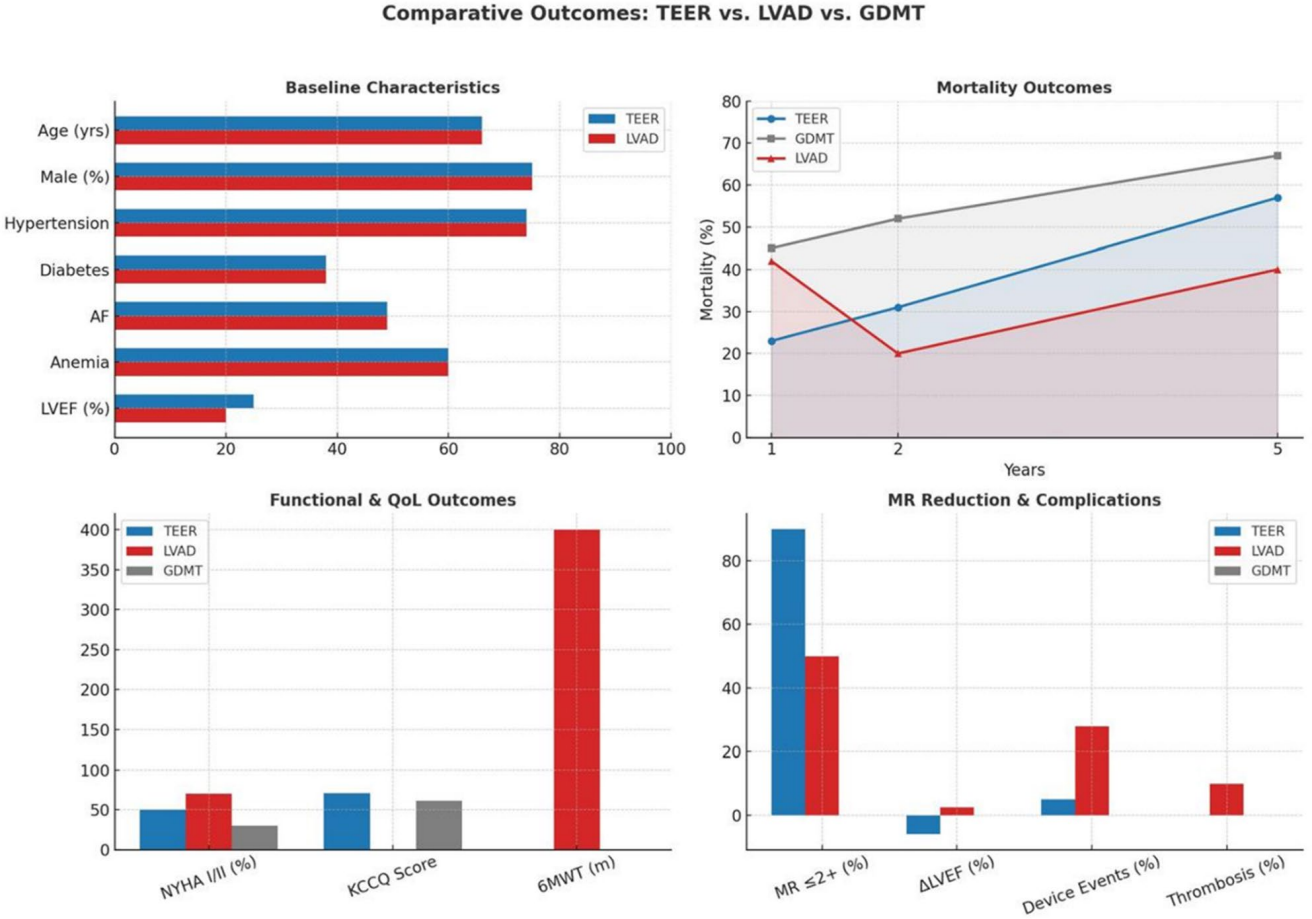
Comparative Outcomes TEER vs. LVAD vs. GDMT

Because this review includes observational studies with heterogeneous populations and study designs, the reported outcomes should be interpreted cautiously. There is limited direct comparative evidence between TEER and LVAD with the majority of data coming from observational studies that are susceptible to selection bias and heterogeneity in disease severity, device generation, and outcome definitions. Studies on TEER usually include patients with more severe MR and less advanced HF, while LVAD series have focused mainly on end-stage disease of different severity of MR [9, 13]. Randomized or propensity-matched comparisons in clinically similar patient populations with the addition of sophisticated imaging modalities, biomarkers, cost-effectiveness analyses and patient-reported outcomes should be a focus for future research. With the advent of newer TEER systems and completely implantable LVADs, it will be crucial to define the optimal timing and sequencing of these strategies to personalize the management of HF (Fig. 2).

## Conclusion

This scoping review suggests that TEER and LVAD play complementary roles in the management of advanced heart failure with secondary mitral regurgitation. TEER is generally considered for patients who remain hemodynamically stable. Conversely, LVAD therapy provides sustained circulatory support to patients with advanced illnesses. There is a need to individualize treatment based on patients’ clinical status due to differences in treatment aims and disease severity. Optimal treatment requires a multidisciplinary heart-team approach that aligns intervention choice with the hemodynamic profile, MR degree, comorbidities, patient goals, and preferences. Overall, evidence gaps in timing, patient selection, and long-term outcomes significantly highlight the need to improve clinical decision-making and patient quality of life.

## Electronic Supplementary Material

Supplementary Material 1

## Data Availability

No datasets were generated or analysed during the current study.

## Declarations

### Artificial intelligence statement

No artificial intelligence tools were used in the design, analysis, or data interpretation of this study. Language editing assistance was limited to grammar and clarity improvements.

## References

[1] Savarese G, Becher PM, Lund LH, Seferovic P, Rosano GMC, Coats AJS. Global burden of heart failure: a comprehensive and updated review of epidemiology. Cardiovasc Res. 2023;118(17):3272–87. 10.1093/cvr/cvac013. 35150240 10.1093/cvr/cvac013

[2] Shah KS, Xu H, Matsouaka RA, Bhatt DL, Heidenreich PA, Hernandez AF, et al. Heart failure with preserved, borderline, and reduced ejection fraction. J Am Coll Cardiol. 2017;70(20):2476–86. 10.1016/j.jacc.2017.08.074. 29141781 10.1016/j.jacc.2017.08.074

[3] Heidenreich PA, Bozkurt B, Aguilar D, Allen LA, Byun JJ, Colvin MM, et al. 2022 AHA/ACC/HFSA guideline for the management of heart failure. Circulation. 2022;145(18):e895–1032. 10.1161/CIR.0000000000001063. 35363499 10.1161/CIR.0000000000001063

[4] Milwidsky A, Mathai SV, Topilsky Y, Jorde UP. Medical therapy for functional mitral regurgitation. Circ Heart Fail. 2022;15(9):e009834. 10.1161/CIRCHEARTFAILURE.122.009834. 10.1161/CIRCHEARTFAILURE.122.009689 35862021

[5] Mehra MR, Naka Y, Uriel N, Goldstein DJ, Cleveland JC Jr, Colombo PC, et al. Two-year outcomes with a magnetically levitated cardiac pump in heart failure. N Engl J Med. 2018;378(15):1386–95. 10.1056/NEJMoa1800866. 29526139 10.1056/NEJMoa1800866

[6] Packer M, Grayburn PA, Lindenfeld J, et al. Effect of mitral valve repair on survival in patients with heart failure and secondary mitral regurgitation. Circulation. 2019;139(1):37–47. 10.1161/CIRCULATIONAHA.118.038620.30586701

[7] Obadia JF, Messika-Zeitoun D, Leurent G, Iung B, Bonnet G, Piriou N, et al. Percutaneous repair or medical treatment for secondary mitral regurgitation. N Engl J Med. 2018;379(24):2297–306. 10.1056/NEJMoa1805374. 30145927 10.1056/NEJMoa1805374

[8] Anker SD, Friede T, von Bardeleben RS, Butler J, Khan MS, Diek M, et al. Transcatheter valve repair in heart failure with moderate to severe mitral regurgitation. N Engl J Med. 2024;391(19):1799–809. 10.1056/NEJMoa2314328. 39216092 10.1056/NEJMoa2314328

[9] American College of Cardiology. Cardiovascular outcomes assessment of the MitraClip percutaneous therapy for heart failure patients with functional mitral regurgitation (COAPT). https://www.acc.org/latest-in-cardiology/clinical-trials/2018/09/21/20/12/coapt 10.1016/j.shj.2024.100333 PMC11403024 39290680

[10] Frea S, Pidello S, Angelini F, Boretto P, Bocchino PP, Melis D, et al. Mitral transcatheter edge-to-edge repair in INTERMACS 3–4 profile patients with severe mitral regurgitation. J Cardiovasc Dev Dis. 2024;11(11):373. 10.3390/jcdd11110373. 39590216 10.3390/jcdd11110373 PMC11595302

[11] Valente S, Sciaccaluga C, Sorini Dini C, Righini FM, Cameli M, Bernazzali S, et al. Left ventricular assist device and transcatheter edge-to-edge mitral valve repair in advanced heart failure: allies or enemies? Front Cardiovasc Med. 2024;10:1327927. 10.3389/fcvm.2023.1327927. 38344214 10.3389/fcvm.2023.1327927 PMC10853372

[12] Stone GW, Abraham WT, Lindenfeld J, Kar S, Grayburn PA, Lim DS, et al. Five-year follow-up after transcatheter repair of secondary mitral regurgitation. N Engl J Med. 2023;388(22):2037–48. 10.1056/NEJMoa2300213. 36876756 10.1056/NEJMoa2300213

[13] Mehra MR, Goldstein DJ, Cleveland JC, Cowger JA, Hall S, Salerno CT, et al. Five-year outcomes in patients with fully magnetically levitated vs axial-flow left ventricular assist devices in the MOMENTUM 3 randomized trial. JAMA. 2022;328(12):1233–42. 10.1001/jama.2022.16197. 36074476 10.1001/jama.2022.16197 PMC9459909

[14] Mehra MR, Goldstein DJ, Uriel N, Cleveland JC Jr, Yuzefpolskaya M, Salerno C, et al. A fully magnetically levitated left ventricular assist device—final report. N Engl J Med. 2019;380(17):1618–27. 10.1056/NEJMoa1900486. 30883052 10.1056/NEJMoa1900486

[15] Uriel N, Colombo PC, Cleveland JC Jr, Long JW, Salerno CT, Goldstein DJ, et al. Hemocompatibility-related outcomes in the MOMENTUM 3 trial at 6 months. Circulation. 2017;135(21):2003–12. 10.1161/CIRCULATIONAHA.117.028303. 28385948 10.1161/CIRCULATIONAHA.117.028303

[16] Kreusser MM, Hamed S, Weber A, Schmack B, Volz MJ, Geis NA, et al. MitraClip implantation followed by insertion of a left ventricular assist device in patients with advanced heart failure. ESC Heart Fail. 2020;7(6):3891–900. 10.1002/ehf2.12982. 33107214 10.1002/ehf2.12982 PMC7754960

[17] Fox H, Gyoten T, Rojas SV, Deutsch MA, Schramm R, Rudolph V, et al. Safety, mortality, and hemodynamic impact of patients with MitraClip undergoing left ventricular assist device implantation. J Cardiovasc Transl Res. 2022;15(3):676–86. 10.1007/s12265-021-10178-w. 34713397 10.1007/s12265-021-10178-w PMC9213377

[18] Stone GW, Lindenfeld J, Abraham WT, Kar S, Lim DS, Mishell JM, et al. Transcatheter mitral-valve repair in patients with heart failure. N Engl J Med. 2018;379(24):2307–18. 10.1056/NEJMoa1806640. 30280640 10.1056/NEJMoa1806640

[19] Kumbhani DJ, Hahn RT, Stone GW. Outcomes of transcatheter edge-to-edge repair in advanced heart failure: a meta-analysis of contemporary studies. JACC Heart Fail. 2022;10(3):181–93. 10.1016/j.jchf.2021.11.012.

[20] Frea S, et al. Long-term outcomes after transcatheter edge-to-edge repair in advanced heart failure. Eur J Heart Fail. 2024;26(1):102–14. 10.1002/ejhf.3084.

[21] Fox H, et al. Centrifugal-flow versus axial-flow LVADs: comparative survival and functional outcomes. J Heart Lung Transpl. 2023;42(6):721–31. 10.1016/j.healun.2023.02.008.

[22] Crespo-Leiro MG, et al. Mechanical circulatory support and mitral regurgitation in heart failure: a comparative perspective. Eur Heart J. 2023;44(9):789–99. 10.1093/eurheartj/ehac757.

[23] Hahn RT, Asch FM, Weissman NJ, et al. Impact of tricuspid regurgitation on clinical outcomes: the COAPT trial. J Am Coll Cardiol. 2020;76(11):1305–14. 10.1016/j.jacc.2020.07.035. 32912445 10.1016/j.jacc.2020.07.035

[24] Pausch J, Bhadra OD, Barten M, Schofer N, Conradi L, Reichenspurner H, Bernhardt A. Results after left ventricular assist device implantation in patients with status post transcatheter edge-to-edge repair. Thorac Cardiovasc Surg. 2023;71(Suppl 1):S1–72. 10.1055/s-0043-1761347.

[25] Khoufi EAA. Outcomes of left ventricular assist devices as destination therapy: a systematic review with meta-analysis. Life. 2025;15(1):53. 10.3390/life15010053. 39859993 10.3390/life15010053 PMC11767145

[26] Kranzl M, Stoiber M, Schaefer AK, et al. Driveline features as risk factor for infection in left ventricular assist devices: meta-analysis and experimental tests. Front Cardiovasc Med. 2021;8:784208. 10.3389/fcvm.2021.784208. 34977190 10.3389/fcvm.2021.784208 PMC8716483

