# Supplementary material for: Transcatheter edge-to-edge repair and left ventricular assist devices for secondary mitral regurgitation in advanced heart failure: a scoping review
Source: J Cardiothorac Surg. 2026 May 7;21:463. doi: 10.1186/s13019-026-04245-z (PMC13321436; doi:10.1186/s13019-026-04245-z)
Supplement: Supplementary file 1 — Supplementary Material 1 [file 13019_2026_4245_MOESM1_ESM.docx]

**Supplementary material: Comparative evidence on LVAD vs TEER**

| **Key for abbreviations used in supplementary material** | |
| --- | --- |
| HFrEF | Heart Failure with Reduced Ejection Fraction |
| TEER | Transcatheter Edge-to-Edge Repair |
| LVAD | Left Ventricular Assist Device |
| GDMT | Guideline-Directed Medical Therapy |
| NT-proBNP | N-terminal pro-B-type natriuretic peptide |
| HTN | Hypertension |
| DM | Diabetes Mellitus |
| CDK | Chronic Kidney Disease |
| AF | Atrial Fibrillation |
| NYHA | New York Heart Association |
| 6MWT | 6 Minute Walk Test |
| EF | Ejection Fraction |
| MR | Mitral Regurgitation |
| LV Volumes | Left Ventricle Volumes |
| RVSP | Right Ventricle Systolic Pressure |
| KCCQ | Kansas City Cardiomyopathy Questionnaire |

**Table S1: Search Strategies by Electronic Database**

| **Database** |  | **Search Item** |
| --- | --- | --- |
| PUBMED | 1 | Ventricular Assist Device [Mesh] |
|  | 2 | Left Ventricular Assist Device |
|  | 3 | LVAD |
|  | 4 | ventricular assist devices |
|  | 5 | mechanical circulatory support |
|  | 6 | 1 OR 2 OR 3 OR 4 OR 5 |
|  | 7 | Transcatheter Edge-to-Edge Repair |
|  | 8 | TEER |
|  | 9 | MitraClip |
|  | 10 | transcatheter mitral valve repair |
|  | 11 | percutaneous mitral valve repair |
|  | 12 | transcatheter mitral repair |
|  | 13 | 7 OR 8 OR 9 OR 10 OR 11 OR 12 |
|  | 14 | Heart Failure [Mesh] |
|  | 15 | heart failure |
|  | 16 | advanced heart failure |
|  | 17 | end-stage heart failure |
|  | 18 | congestive heart failure |
|  | 19 | 14 OR 15 OR 16 OR 17 OR 18 |
|  | 20 | Mitral Valve Insufficiency [Mesh] |
|  | 21 | mitral regurgitation |
|  | 22 | functional mitral regurgitation |
|  | 23 | 20 OR 21 OR 22 |
|  | 24 | 6 AND 13 AND 19 AND 23 |
| SCOPUS | 25 | ventricular assist device |
|  | 26 | Left Ventricular Assist Device |
|  | 27 | LVAD |
|  | 28 | 25 OR 26 OR 27 |
|  | 29 | transcatheter edge-to-edge repair |
|  | 30 | TEER |
|  | 31 | MitraClip |
|  | 32 | transcatheter mitral valve repair |
|  | 33 | 29 OR 30 OR 31 OR 32 |
|  | 34 | heart failure |
|  | 35 | advanced heart failure |
|  | 36 | end-stage heart failure |
|  | 37 | congestive heart failure |
|  | 38 | 34 OR 35 OR 36 OR 37 |
|  | 39 | mitral regurgitation |
|  | 40 | functional mitral regurgitation |
|  | 41 | Mitral Valve Insufficiency |
|  | 42 | 39 OR 40 OR 41 |
|  | 43 | 28 AND 33 AND 38 AND 42 |
| EMBASE | 44 | ventricular assist device |
|  | 45 | Left Ventricular Assist Device |
|  | 46 | LVAD |
|  | 47 | 44 OR 45 OR 46 |
|  | 48 | heart failure |
|  | 49 | advanced heart failure |
|  | 50 | end-stage heart failure |
|  | 51 | 48 OR 49 OR 50 |
|  | 52 | mitral regurgitation |
|  | 53 | functional mitral regurgitation |
|  | 54 | Mitral Valve Insufficiency |
|  | 55 | 52 OR 53 OR 54 |
|  | 56 | transcatheter edge-to-edge repair |
|  | 57 | TEER |
|  | 58 | MitraClip |
|  | 59 | transcatheter mitral valve repair |
|  | 60 | 56 OR 57 OR 58 OR 59 |
|  | 61 | Observational |
|  | 62 | Cohort |
|  | 63 | 'case control |
|  | 64 | Registry |
|  | 65 | Retrospective |
|  | 66 | Prospective |
|  | 67 | 61 OR 62 OR 63 OR 64 OR 65 OR 66 |
|  | 68 | 47 AND 51 AND 55 AND 60 AND 67 |
| GOOGLE SCHOLAR | 69 | Left Ventricular Assist Device |
|  | 70 | LVAD |
|  | 71 | 69 OR 70 |
|  | 72 | transcatheter edge-to-edge repair |
|  | 73 | TEER |
|  | 74 | MitraClip |
|  | 75 | transcatheter mitral valve repair |
|  | 76 | 72 OR 73 OR 74 OR 75 |
|  | 77 | mitral regurgitation |
|  | 78 | functional mitral regurgitation |
|  | 79 | 77 OR 78 |
|  | 80 | heart failure |
|  | 81 | advanced heart failure |
|  | 82 | end-stage heart failure |
|  | 83 | 80 OR 81 OR 82 |
|  | 84 | 71 AND 76 AND 79 AND 83 |

**Table S2: Inclusion and Exclusion Criteria**

|  | **Inclusion Criteria** | **Exclusion Criteria** |
| --- | --- | --- |
| **Study design** | Observational study designs (retrospective/prospective cohorts, registry studies, case-control).  Studies published in English. | Case reports, case series (<10 patients), editorials, reviews, conference abstracts without data. Animal or preclinical studies.  Randomized controlled trials (if restricted to observational evidence only).  Non-English studies. |
| **Participants** | Adult patients (≥18 years) with the diagnosis of advanced HFrEF with functional MR. | Pediatric or congenital heart disease populations. |
| **Intervention** | Left Ventricular Assist Device (LVAD) implantation (any type: durable continuous-flow devices, bridge-to-transplant, destination therapy). |  |
| **Comparator** | Transcatheter Edge-to-Edge Repair (TEER) of the mitral valve (e.g., MitraClip or similar devices). |  |
